# Unidirectional Synchronization of Hodgkin-Huxley Neurons With Prescribed Performance Under Transcranial Magneto-Acoustical Simulation

**DOI:** 10.3389/fnins.2019.01061

**Published:** 2019-10-15

**Authors:** Dan Liu, Song Zhao, Xiaoyuan Luo, Yi Yuan

**Affiliations:** ^1^School of Electrical Engineering, Yanshan University, Qinhuangdao, China; ^2^College of Integrative Medicine, Hebei University of Chinese Medicine, Shijiazhuang, China; ^3^Department of Medical Imaging, The Second Hospital of Hebei Medical University, Shijiazhuang, China

**Keywords:** TMAS, Hodgkin-Huxley neuron, unidirectional synchronization, adaptive neural control, predefined performance

## Abstract

This paper exploits the unidirectional synchronization dynamics of two Hodgkin-Huxley (HH) neurons under transcranial magneto-acoustical stimulation (TMAS). The major purpose is to explore a control scheme to make the spiking modes of the neural potentials stimulated by TMAS achieve synchronization states under the feedback input. For this purpose, an adaptive neural controller, which makes the neurons satisfy the prescribed master-slaver synchronization performance, is designed by introducing a tracking error into Lyapunov analysis. Under the proposed control scheme, the slaver neuron can not only overcome the model uncertainties and the difficulties brought by prescribed performance, but also track the spiking patterns of the master neuron. Finally, the simulations are implemented to demonstrate the effectiveness of the proposed controller, that is, the TMAS induced synchronization states of the HH neuron system can achieve the prescribed performance under the proposed controller.

## Introduction

Transcranial magneto-acoustical stimulation (TMAS), as a new technology for brain stimulation, has many advantages in space resolution and penetration depth. Previous studies demonstrated that TMAS can alter neuronal firing rhythm, phase-locking and concentration of Ca^2+^ (Norton, 2003; Yi et al., 2017). However, few studies investigate the synchronization control for neuronal activity induced by TMAS. In recent decades, many scholars have made effort to lucubrate the biological information processing in neuroscience (Gray et al., 1989; Meister et al., 1991). Experimental reports indicate that the synchronization activities of neurons have a significant effect on thinking, motion control, and diseases, such as Parkinson's, Huntington's, and epilepsy (Gray, 1994; Niebur et al., 2002; Fries, 2005; Hammond et al., 2007), and so on.

From a neuroscientific viewpoint, there are two kinds of synchronization methods (Boccaletti et al., 2002). One is the natural approach of diffusive coupling and intrinsic noise. This approach is considered as the initiator of nervous activity to study the synchronization dynamics (Wang, 2002; Acker et al., 2003; Casado, 2003). The other is investigated from control engineering. There are two common ways used for synchronization of dissimilar neurons. One is observer-based synchronization (Boccaletti et al., 2002), which designs state observer to make the nonlinear oscillators synchronize. The other is controller-based synchronization (Deng et al., 2006; Wang et al., 2007a,b; Aguilar-López and Martínez-Guerra, 2008; Sisi et al., 2009; Che et al., 2011; Yu et al., 2013), which uses a control scheme to realize the synchronization.

There are several challenges in synchronization control for different neurons due to the characteristics of neuron systems, such as the presence of disturbance, dynamic uncertainty, and nonlinearity in neuronal models. These challenges enthuse researchers to devote themselves to this research issue, and many effective methods have been presented.

In order to overcome perturbations, sliding-mode control laws were proposed between two coupled neurons (Aguilar-López and Martínez-Guerra, 2008; Che et al., 2011; Yu et al., 2013). Deng et al. introduced a backstepping control scheme on account of Lyapunov analysis to achieve synchronization in spite of external disturbances (Deng et al., 2006). Based on feedback linearization theories, nonlinear controllers were introduced to reach synchronization of coupled neurons in spite of unmeasured states (Octavio Cornejo-Pérez, 2005; Wang et al., 2007a,b). Le and Hong presented nonlinear and linear parameter adaptation controllers to overcome system uncertainties and achieve synchronization of two coupled neurons (Le and Hong, 2013). Robust control schemes, combining linear matrix inequality with parameter adaptation to deal with system uncertainty and reach synchronization, were proposed by Rehan and Hong (2011). Puebla et al. designed a robust control scheme for two coupled neurons, which has the uncertainty compensation and error tracking functions (Puebla et al., 2010). Wang and Zhao introduced a system dynamics inversion into nonlinear controller to ensure the neuron synchronization under system uncertainty (Wang and Zhao, 2010). Synchronization of two coupled direction-dependent neurons with unknown and uncertain parameters was discussed in Iqbal et al. (2014). Some conclusions for the adaptive hybrid chaotic synchronization of the identical neuron models, using Lyapunov stability theory, were proposed (Aqil et al., 2012; Baladron et al., 2012; Vaidyanathan, 2015). More recently, a novel robust synchronization approach based on the master-slave configuration was introduced for neuronal systems (Puebla et al., 2017), and an adaptive feedback control law was designed for synchronization of the coupled neurons with a time delay (Iqbal et al., 2018). Despite the synchronization control of coupled neurons has been widely investigated, only the steady state synchronization performance has been focused on. Scholars have long recognized transient state control performance worth in-depth study.

The prescribed performance control (PPC) of synchronization means that the tracking error should converge to a predefined arbitrarily small residual set with convergence rate being no less than a prescribed value, that is, exhibiting maximum overshoot is less than a sufficiently small preassigned constant (Kelly, 1995). In order to enhance the control effect, a great deal of researches focusing on PPC have been developed in various control fields, such as robotic systems (Bechlioulis and Rovithakis, 2009; Bechlioulis et al., 2010), multi-agent systems (Karayiannidis and Doulgeri, 2012), and teleoperation systems (Yang et al., 2015). In unidirectional synchronization control for different neurons, the trajectory of a slaver neuron must track, to some extent, the trajectory of a master neuron even if the slaver and master system is dissimilar (Octavio Cornejo-Pérez, 2005). Better transient-state control performance means better tracking effect, and further, better therapeutic effect in neuroscience. However, as far as we know, the study of prescribed performance synchronization control of neuron systems with TMAS has not been launched.

Mathematical modeling has had a great influence on neuroscience (De Schutter and Ebrary, 2010). There are three common models of neuron systems to study neuron synchronization control (Fitzhugh, 1961; Hodgkin and Huxley, 1989; Girardi-Schappo et al., 2013). The most common model in biology is the Hodgkin-Huxley (HH) neuron model, which is used as a realistic neuron model in the synchronization research of neural systems. The FitzHugh-Nagumo (FHN) neuron model is another famous model, which can be seen as a simplified approximation of the HH neuron model by reserving main characteristics of its action potential. Another common model is the Hindmarsh-Rose (HR) neuron model representing physiological transmission of neural signal. The HR model can be seen as a reduction type of the HH model in neurobiology. Compared with these simplified neuron models, the HH model shows us in detail how the neuron action potentials are excited, and how the potentials approximately express the electric properties of excitable cells. Thus, in the available literatures, few researchers focused on the synchronization of the HH neurons as its complexity.

In this study, we propose an adaptive neural synchronization control scheme for two HH neurons under TMAS. The designed control law is endowed with ensuring the prescribed synchronization performance of the neuronal system. To restrict the tracking error, a synchronization error transformation is constructed to convert the restricted synchronization control problem to an unrestricted one. Besides, the Radial Basic Function neural network (RBF NN) is introduced to overcome the system uncertainty. Finally, a stable synchronization control scheme which ensures the prescribed neural tracking performance is proposed.

The main contributions in this work are: (i) The PPC is introduced into synchronization control of neuron system, which can enhance the synchronization effect. (ii) The neurons are exposed to external transcranial magneto-acoustical stimuli. The TMAS, a novel brain stimulation technology, has been applied for neurological and psychiatric disease treatment and rehabilitation. (iii) The RBFNN is applied to overcome the system uncertainty, and a filtered synchronization error is constructed for designing the stable neural control law.

The remainder of this work is organized as follows. In section Methods, we introduce the problem formulation and preliminaries. Prescribed performance control scheme is introduced to guarantee the synchronization constraint and stability of the closed-loop system. The main results in simulation studies and its improved effects are shown in section Results. Section Discussion concludes the obtained results and the existing barriers, as well as looks for future research directions.

## Methods

### HH Neuron Model

**Figure 1:**
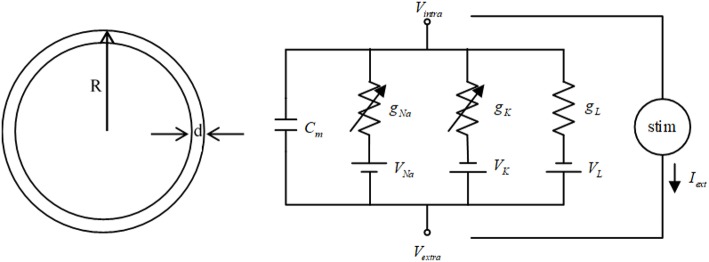
The cell model and the diagram of equivalent circuit of the HH neuron model.

The HH neuron model (Hodgkin and Huxley, 1989) is used in this study. The cell model and the diagram of equivalent circuit of the HH neuron model are shown in Figure 1. In the cell model, the cell radius is *R* = 10 μm, and the thickness of cell membrane is *d* = 0.5 nm (Che et al., 2012). The HH neuron model can be represented by the following nonlinear equations (Che et al., 2012).

(1) $$\begin{array}{l} \begin{array}{c} C\frac{dV}{dt}=I_{ext}-g_{K}n^{4}\left(V-V_{K}\right)-g_{Na}m^{3}h\left(V-V_{Na}\right)\\ \;-g_{L}\left(V-V_{L}\right)\\ \frac{dn}{dt}\;=\alpha_{n}\left(V\right)\left(1-n\right)-\beta_{n}\left(V\right)n\\ \frac{dm}{dt}\;=\alpha_{m}\left(V\right)\left(1-m\right)-\beta_{m}\left(V\right)m\\ \frac{dh}{dt}\;=\alpha_{h}\left(V\right)\left(1-h\right)-\beta_{h}\left(V\right)h \end{array} \end{array}$$

In Equation (1), *V* is the membrane potential of HH neuron. *n* represents the activation of the *K*^+^ current. *m* and *h* are the gating variables of the activation and inactivation of the *Na*^+^ current, respectively. *C* is the membrane capacitance. *V*_*K*_, *V*_*Na*_, and *V*_*L*_ are the equilibrium potentials of the sodium, the potassium and the leak electric currents, respectively. *g*_*K*_, *g*_*Na*_, and *g*_*L*_ are the maximal conductance of the corresponding ionic electric currents. The external stimulus term *I*_*ext*_ can be modeled by the external current generated by TMAS. The explicit forms of the equations α_*j*_(*V*) and β_*j*_(*V*) (*j* = *n, m, h*) in Equation (1) are given as follows.

(2) $$\begin{array}{l} \begin{array}{c} \alpha_{n}\left(V\right)=0.1\left(100-V\right)/\left[\exp\left(\left(100-V\right)/100\right)-1\right]\\ \beta_{n}\left(V\right)=0.15\exp\left(-V/100\right)\\ \alpha_{m}\left(V\right)=0.1\left(15-V\right)/\left[\exp\left(\left(15-V\right)/5\right)-1\right]\\ \beta_{m}\left(V\right)=4\exp\left(-V/10\right)\\ \alpha_{h}\left(V\right)=0.08\exp\left(-V/10\right)\\ \beta_{h}\left(V\right)=1/\left[\exp\left(\left(-V+30\right)/10\right)+1\right] \end{array} \end{array}$$

The master and slaver HH neuron system is redefined by Equation (3) and Equation (4) to state the synchronization problem. *x*_*M,i*_ and *x*_*S,i*_(*i* = 1, 2, 3, 4) represent the states *V*, *n*, *m*, and *h* in the master and slaver system. By considering the essential characteristics of the synchronous behavior of two neurons, the master HH neuron system is proposed as follows.

(3) $$\begin{array}{l} {\dot{x}}_{M,1}=\frac{1}{C_{M}}(I_{ext\_M}-g_{KM}x_{M,2}^{4}\left(x_{M,1}-V_{KM}\right)\\ -g_{NaM}x_{M,3}^{3}x_{M,4}\left(x_{M,1}-V_{NaM}\right)-g_{LM}\left(x_{M,1}-V_{LM}\right))\\ {\dot{x}}_{M,2}=\alpha_{n}\left(x_{M,1}\right)\left(1-x_{M,2}\right)-\beta_{n}\left(x_{M,1}\right)x_{M,2}\\ {\dot{x}}_{M,3}=\alpha_{m}\left(x_{M,1}\right)\left(1-x_{M,3}\right)-\beta_{m}\left(x_{M,1}\right)x_{M,3}\\ {\dot{x}}_{M,4}=\alpha_{h}\left(x_{M,1}\right)\left(1-x_{M,4}\right)-\beta_{h}\left(x_{M,1}\right)x_{M,4} \end{array}$$

and the slaver HH neuron system is denoted by the following equations:

(4) $$\begin{array}{l} {\dot{x}}_{S,1}=\frac{1}{C_{M}}(I_{ext\_S}-g_{KS}x_{S,2}^{4}\left(x_{S,1}-V_{KS}\right)\\ -g_{NaS}x_{S,3}^{3}x_{S,4}\left(x_{S,1}-V_{NaS}\right)-g_{LS}\left(x_{S,1}-V_{LS}\right))+u\\ {\dot{x}}_{S,2}=\alpha_{n}\left(x_{S,1}\right)\left(1-x_{S,2}\right)-\beta_{n}\left(x_{S,1}\right)x_{S,2}\\ {\dot{x}}_{S,3}=\alpha_{m}\left(x_{S,1}\right)\left(1-x_{S,3}\right)-\beta_{m}\left(x_{S,1}\right)x_{S,3}\\ {\dot{x}}_{S,4}=\alpha_{h}\left(x_{S,1}\right)\left(1-x_{S,4}\right)-\beta_{h}\left(x_{S,1}\right)x_{S,4} \end{array}$$

where the item *u* is the feedback control input. Table 1 shows the fixed parameters of the master-slaver HH neuronal system. Under some parameters, both the two neurons cannot be synchronous originally.

**Table 1:** Fixed parameters of the master-slaver HH neuronal system.

**Parameters of the master neuron**	**Parameters of the slaver neuron**
*C*_*M*_ = 1 μ*F*/cm^2^	*C*_*S*_ = 0.9 μ*F*/cm^2^
$g_{KM}=36\;mS/\text{c}{\text{m}}^{2}$	$g_{KS}=32.4\;mS/\text{c}{\text{m}}^{2}$
$g_{NaM}=120\;mS/\text{c}{\text{m}}^{2}$	$g_{NaS}=108\;mS/\text{c}{\text{m}}^{2}$
$g_{LM}=0.3\text{ mS}/\text{c}{\text{m}}^{2}$	$g_{LS}=0.27\;mS/\text{c}{\text{m}}^{2}$
*V*_*KM*_ = 12 mV	*V*_*KS*_ = 10.8 mV
*V*_*NaM*_ = −115 mV	*V*_*NaS*_ = −103.5 mV
*V*_*LM*_ = −10.613 mV	*V*_*LM*_ = −9.5517 mV

### Principle of TMAS

TMAS, as a novel brain stimulation technology, generates a safe electric current to noninvasively stimulate the nervous tissue. The main principle of TMAS is to integrate ultrasound waves with a static magnetic field to produce the suitable stimulation current (Norton, 2003; Yuan et al., 2016). In TMAS, an ultrasonic wave spreads in electrolytic fluid and makes the ions move with it. In a static magnetic field, the moving ions will produce a Lorentz force. This force then generates an electric current that oscillates with the fundamental and modulation frequency (Ammari et al., 2015; Graslandmongrain et al., 2015). In (Yuan et al., 2016), the relationship between the generated electric current and the ultrasonic and magnetic field parameters has been deduced, and it can be represented by

(5) $$\begin{array}{l} J_{y}=\sigma B_{x}\sqrt{\frac{2\Gamma }{\rho c_{0}}}\sin\left(2\pi ft\right) \end{array}$$

*J*_*y*_ is the electric current density that is equivalent to the External stimulus current *I*_*ert*_. σ is the conductivity, and its typical value is 0.5 Siemens/m (Norton, 2003). *B*_*x*_, Γ, ρ, *c*_0_, and *f* are magnetic field intensity, ultrasonic intensity, tissue density, ultrasound speed, and ultrasonic fundamental frequency, respectively. The fixed parameters for Equation (5) are listed in Table 2. In this work, the electrical current *I*_*ert*_, induced by ultrasound and magnetic field in brain tissue, is used as the external current to drive the cross membrane current and alter the membrane potentials of neurons in Hodgkin-Huxley model.

**Table 2:** Fixed parameters for TMAS.

**Parameters**	**Value**	**Unit**
σ	0.5	Siemens/m
*B*_*x*_	7	Teslas
Γ	100	Watt/cm^2^
ρ	1120	Kg/m^3^
*c*_0_	1540	m/s
*f*	200	Hz

**Figure 2:**
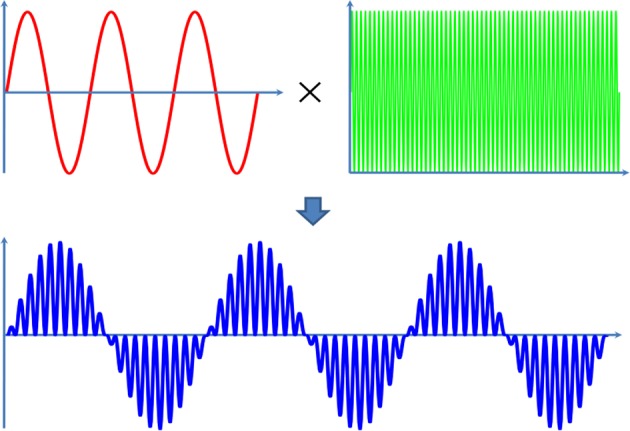
The schematic of the ultrasound frequency in TMAS.

In this study, a pulsed ultrasound, modulated by a sine wave and a continuous wave, is applied (Figure 2). The modulation ultrasound can be described by the following equation and the modulation frequency (*MF*) is ranged from 100 to 120 Hz.

(6) $$\begin{array}{l} \sin\left(2\pi \left(MF\right)t\right)\times \left(\sin\left(2\pi ft\right)+1\right) \end{array}$$

Thus, the generated electric current *I*_*ert*_ which is utilized to stimulate the neurons, can be described by.

(7) $$\begin{array}{l} I_{ext}=\sigma B_{x}\sqrt{\frac{2\Gamma }{\rho c_{0}}}\left(\sin\left(2\pi \left(MF\right)t\right)\times \left(\sin\left(2\pi ft\right)+1\right)\right) \end{array}$$

### Properties of RBF Neural Networks

The RBF neural network is used widely due to its linearly parameterized structure, and can be described by:

(8) $$\begin{array}{l} h_{nn}\left(Z\right)=\overset{l}{\underset{i=1}{\sum }}\omega_{i}s_{i}\left(Z\right)=W^{T}S\left(Z\right) \end{array}$$

where *W* ∈ *R*^*q*×*l*^ is weight vector, $Z\in \Omega_{z}\subset R^{q}$ is input vector, and *l* is the NN node number. $S\left(Z\right)={\left[s_{1}\left(Z\right),s_{2}\left(Z\right),\ldots ,s_{l}\left(Z\right)\right]}^{T}$ is a vector of Gaussian basis functions, of which the commonly used expression is $s_{i}\left(Z\right)=\exp\left[-{\left(Z-\mu_{i}\right)}^{T}\left(Z-\mu_{i}\right)/\eta_{i}^{2}\right]$, *i* = 1, 2, …, *l*, where μ_*i*_ are constant vectors called the center of the receptive field and η_*i*_ is a real number called the width of the basis function. The approximation property in Loría and Panteley (2002) indicates that, for some sufficiently large integer *l*, *W*^*T*^*S*(*Z*) can approximate any given function with the approximation error restricted by δ^*^, i.e.,

(9) $$\begin{array}{l} h_{nn}\left(Z\right)={W^{*}}^{T}S\left(Z\right)+\delta \left(Z\right),\forall Z\in \Omega_{z}\subset R^{q} \end{array}$$

where W^*^ is the ideal constant weight vector, and δ(*Z*) is the approximate error restricted to |δ(*Z*)| < δ^*^ with constant δ^*^ > 0 for all ∀*Z* ∈ Ω_*z*_. W^*^ is defined as the ideal value of *W* that minimizes |δ(*Z*)| for all $Z\in \Omega_{z}\subset R^{q}$, i.e., $W^{*}=\arg\min_{W\subset R^{l}}\left\{\sup_{Z\subset \Omega_{z}}|h_{nn}\left(Z\right)-W^{T}S\left(Z\right)\right\}$. The desired weight W^*^ needs to be estimated in the process of controller design.

Before proposing our main conclusions, the following assistant lemma should be firstly introduced.

***Lemma 1*** (Yang et al., 2016). ∀(*x, y*) ∈ *R*^2^, the following inequality holds

(10) $$\begin{array}{l} xy\leq \frac{\varsigma^{p}}{p}{\left|x\right|}^{p}+\frac{1}{q\varsigma^{q}}{\left|y\right|}^{q} \end{array}$$

where ς > 0, *p* > 1, *q* > 1 and (*p* − 1)(*q* − 1) = 1.

### General Model of Synchronized Potential

To transform the problem formulation into a synchronization model, we consider a more common class of master-slaver configuration of neurons connected with membrane potential than the neuronal systems (3) and (4). According to Equation (3), the dynamics of the master neuron can be reformulated by

(11) $$\begin{array}{l} \begin{array}{c} {\dot{x}}_{M,1}=f_{M,1}\left(x_{M}\right)\\ {\dot{x}}_{M,j}=f_{M,j}\left(x_{M}\right) \end{array} \end{array}$$

where *x*_*M*,1_ represents membrane potential of the master neuron, *x*_*M,j*_, *j* = 2, 3, 4 are the remaining state variables of Equation (3) and $x_{M}={\left[x_{M,1},x_{M,2},x_{M,3},x_{M,4}\right]}^{T}$.

According to Equation (4), the dynamics of the slaver neuron can be reformulated by

(12) $$\begin{array}{l} \begin{array}{c} {\dot{x}}_{S,1}=f_{S,1}\left(x_{S}\right)+u\\ {\dot{x}}_{S,j}=f_{S,j}\left(x_{S}\right) \end{array} \end{array}$$

where *x*_*S*,1_ represents membrane potential of the slaver neuron, *x*_*S,j*_, *j* = 2, 3, 4 are the remaining state variables of Equation (4) and $x_{S}={\left[x_{S,1},x_{S,2},x_{S,3},x_{S,4}\right]}^{T}$. *u* is an external input applied to the slaver neuron, which represents a feedback synchronization force.

The synchronization error system for the HH neuronal system can be modeled as

(13) $$\begin{array}{l} \dot{e}={\dot{x}}_{M,1}-{\dot{x}}_{S,1}=f_{S,1}\left(x_{S}\right)-f_{M,1}\left(x_{M}\right)+u=f\left(x\right)+u \end{array}$$

where *e* = *x*_*M*,1_ − *x*_*S*,1_ denotes the synchronization error, and $x={\left[x_{M},x_{S}\right]}^{T}$.

### Prescribed Performance Control

In this section, an adaptive neural controller, which guarantees the prescribed transient and steady tracking performance for the master-slaver neuron system, is designed by integrating the prescribed performance function to the constructed transformation error system.

The prescribed performance generally includes the minimum convergence rate and the maximum steady state error. In addition, allowable overshoot needs to be bounded by a decaying function of time as a priori. Prescribed synchronization performance is accomplished if all the elements of the tracking error *e* evolve strictly within the arbitrarily small predefined region. We can use the following mathematical expressions to express the prescribed performance:

(14) $$\begin{array}{l} -H\beta \left(t\right)<e<\beta \left(t\right),\;if\;e\left(0\right)\geq 0 \end{array}$$

(15) $$\begin{array}{l} -\beta \left(t\right)<e<H\beta \left(t\right),\;if\;e\left(0\right)\leq 0 \end{array}$$

where 0 < *H* ≤ 1, β (*t*) is a performance function and can be defined in our work as

(16) $$\begin{array}{l} \beta \left(t\right)=\left(\beta_{0}-\beta_{\infty }\right)e^{-\kappa t}+\beta_{\infty } \end{array}$$

where β_0_, β_∞_, and κ are strictly positive constants. β_0_ = β(0) and β_∞_ = lim_*t*→∞_ β (*t*). From (14) and (15), the constant κ denotes the minimal convergence rate, the β_∞_ is the maximal steady state error, and the maximum overshoot is less than *Hβ*_0_.

In order to constrain the tracking error to the prescribed range, an error transformation is introduced to make an equivalent unconstrained tracking error condition instead of the constrained one. We then define the following state transformation:

(17) $$\begin{array}{l} e\left(t\right)=\beta \left(t\right)R\left(\varepsilon \right) \end{array}$$

where ε(*t*) is the filtered tracking error and *R*(▪) is a smooth and strictly increasing function defining a bijective mapping

(18) $$\begin{array}{l} R:\left(-H,1\right)\to \left(-\infty ,+\infty \right),\;if\;e\left(0\right)\geq 0 \end{array}$$

(19) $$\begin{array}{l} R:\left(-1,H\right)\to \left(-\infty ,+\infty \right),\;if\;e\left(0\right)\leq 0 \end{array}$$

We employ the following smooth and increasing function as *R*(ε)

(20) $$\begin{array}{l} R\left(\varepsilon \right)=\frac{e^{\varepsilon }-H}{1+e^{\varepsilon }},\;if\;e\left(0\right)\geq 0 \end{array}$$

(21) $$\begin{array}{l} R\left(\varepsilon \right)=\frac{He^{\varepsilon }-1}{1+e^{\varepsilon }},\;if\;e\left(0\right)\leq 0 \end{array}$$

Then the filtered tracking error ε can be represented by

(22) $$\begin{array}{l} \varepsilon =R^{-1}\left(e/\beta \left(t\right)\right)=\ln\;\left[\left(H+e/\beta \left(t\right)\right)/\left(1-e/\beta \left(t\right)\right)\right],\\ \;if\;e\left(0\right)\geq 0 \end{array}$$

(23) $$\begin{array}{l} \varepsilon =R^{-1}\left(e/\beta \left(t\right)\right)=\ln\;\left[\left(1+e/\beta \left(t\right)\right)/\left(H-e/\beta \left(t\right)\right)\right],\\ \;if\;e\left(0\right)\leq 0 \end{array}$$

The derivative of the filtered error is

(24) $$\begin{array}{l} \dot{\varepsilon }=\varphi \left(\dot{e}-\frac{\dot{\beta }\left(t\right)}{\beta \left(t\right)}e\right) \end{array}$$

where ė = *f*(*x*) + *u*, and

(25) $$\begin{array}{l} \varphi =\frac{1}{H\beta \left(t\right)+e}-\frac{1}{e-\beta \left(t\right)},\;if\;e\left(0\right)\geq 0 \end{array}$$

(26) $$\begin{array}{l} \varphi =\frac{1}{\beta \left(t\right)+e}-\frac{1}{e-H\beta \left(t\right)},\;if\;e\left(0\right)\leq 0 \end{array}$$

From (14) and (15), we know ϕ > 0. The derivative of the filtered error (24) can be rewritten as

(27) $$\begin{array}{l} \dot{\varepsilon }=\varphi \left(\dot{e}-\frac{\dot{\beta }\left(t\right)}{\beta \left(t\right)}e\right)=\varphi \left(f\left(x\right)+u\right)+\gamma \left(e,t\right) \end{array}$$

where

(28) $$\begin{array}{l} \gamma =\varphi \frac{\dot{\beta }\left(t\right)}{\beta \left(t\right)}e \end{array}$$

Since *f*(*x*) in (27) is smooth and unknown, a RBFNN *W*^*T*^*S*(*Z*) is used to approach *f*(*x*). With the above definitions, the neural controller is designed by

(29) $$\begin{array}{l} u=-k\left(t\right)\varepsilon -{\hat{W}}^{T}S\left(Z\right)-\gamma /\varphi \end{array}$$

where Ŵ is the estimation of the unknown ideal weight vector, and the control gain *k*(*t*) satisfies $k\left(t\right)=k_{1}+k_{2}-\dot{\varphi }/2\varphi^{2}$with *k*_1_ and *k*_2_ being positive constants.

The adaptive updating law of Ŵ is given by

(30) $$\begin{array}{l} \dot{\hat{W}}=P\left[S\left(x\right)\varepsilon -\xi \hat{W}\right] \end{array}$$

where *P* = *P*^*T*^ > 0 and ξ > 0 is designed parameter.

***Remark 1***. The initial synchronization error *e*(0) should satisfy *e*(0) < β(0), which is confined by the prescribed performance condition (14) and (15). With this restriction, the restricted synchronization control of the neuron system (4) is equivalently converted to the stability problem.

### Analysis of Performance

The main conclusions of our study are exhibited in this section. Furthermore, the stability and the membrane potential synchronization performance are proven by Lyapunov method.

***Theorem 1***. Considering the master-slaver HH neuron system being composed of the master reference system (3) and the slaver-controlled system (4), under the adaptive neural controller (29) with the filter tracking error (22) and (23) and the NN weight updated laws (30), the master-slaver HH neuron system can be achieved the synchronization configuration constrained by the prescribed performance (14) and (15).

***Proof***: We construct the following Lyapunov function

(31) $$\begin{array}{l} V=\frac{1}{2}\frac{\varepsilon^{2}}{\varphi }+\frac{1}{2}{\tilde{W}}^{T}Q^{-1}\tilde{W} \end{array}$$

where $\tilde{W}=Ŵ-W^{*}$ is the estimation error vector.

Based on the definition in section Prescribed Performance Control, one has

(32) $$\begin{array}{l} \dot{\varepsilon }=\varphi \left({W^{*}}^{T}S\left(x\right)+\delta \left(x\right)+u+\frac{\gamma }{\varphi }\right) \end{array}$$

Substituting (29) into (32), one yields

(33) $$\begin{array}{l} \dot{\varepsilon }=\varphi \left(-k\left(t\right)\varepsilon -{\tilde{W}}^{T}S\left(x\right)+\delta \left(x\right)\right) \end{array}$$

From (30) and (33), we can get the time derivative of *V* as

(34) $$\begin{array}{c} \dot{V}=\varepsilon \left(-k\left(t\right)\varepsilon -{\tilde{W}}^{T}S\left(x\right)+\delta \left(x\right)\right)-\frac{\dot{\varphi }}{2\varphi^{2}}\varepsilon^{2}+{\tilde{W}}^{T}Q^{-1}\dot{\hat{W}}\\ \;=-k\left(t\right)\varepsilon^{2}-\frac{\dot{\varphi }}{2\varphi^{2}}\varepsilon^{2}+\varepsilon \delta \left(x\right)-{\tilde{W}}^{T}Q^{-1}\left(\dot{\hat{W}}-PS\left(x\right)\varepsilon \right) \end{array}$$

Substituting (30) into (34), one has

(35) $$\begin{array}{l} \dot{V}=-k\left(t\right)\varepsilon^{2}-\frac{\dot{\varphi }}{2\varphi^{2}}\varepsilon^{2}+\varepsilon \delta \left(x\right)-{\tilde{W}}^{T}\xi \hat{W} \end{array}$$

According to Lemma 1, one can obtain

(36) $$\begin{array}{l} \varepsilon \delta \left(x\right)\leq \frac{{\delta^{*}}^{2}}{4k_{1}}+k_{1}\varepsilon^{2} \end{array}$$

and

(37) $$\begin{array}{l} -{\tilde{W}}^{T}\xi \hat{W}\leq \frac{{\tilde{W}}^{T}\xi \tilde{W}}{2}+\frac{\xi {\left||W^{*}|\right|}^{2}}{2} \end{array}$$

Then, one can further get

(38) $$\begin{array}{l} \dot{V}\leq -k_{2}\varepsilon^{2}-\frac{{\tilde{W}}^{T}\xi \hat{W}}{2}+b\leq -aV+b \end{array}$$

where $a=\min\left\{2k_{2}\varphi ,\frac{2\xi }{\lambda_{\max}\left(Q^{-1}\right)}\right\}$, and $b=\frac{\xi {\left||W^{*}|\right|}^{2}}{2}+\frac{{\delta^{*}}^{2}}{4k_{1}}$.

Let θ = *b*/*a*, it can be further deduced that

(39) $$\begin{array}{l} \dot{V}\leq \left(V\left(0\right)-\theta \right)\exp\left(-at\right)+\theta \end{array}$$

which stands for

(40) $$\begin{array}{l} |\varepsilon |\leq \sqrt{2\varphi \theta } \end{array}$$

as *t* → ∞. From (31), (39), and (40), the filter error ε and the weight error $\tilde{W}$ are proven to be bounded. It is then obvious that Ŵ is bounded since $\tilde{W}=Ŵ-W^{*}$ and W^*^ is the desired constant. According to the error transformation Equation (17), the neuron synchronization error *e* converges to the predefined small neighborhood of the zero, and the convergence rate and overshoot likewise satisfy the prescribed performance, which is represented by performance function β(*t*) and the parameter *H*. The proof is completed.

***Remark 2***. It should be noticed that the different choices of the controller parameters have different influences on the synchronization performance. From (38)–(40), we can find that the convergence region of the filter error ε can be reduced by choosing a large *k*_1_, a big *Q* or *k*_2_ and a small ξ. Besides, if the approximation error δ of the RBFNN is small enough, the same effect can be achieved.

## Results

**Figure 3:**
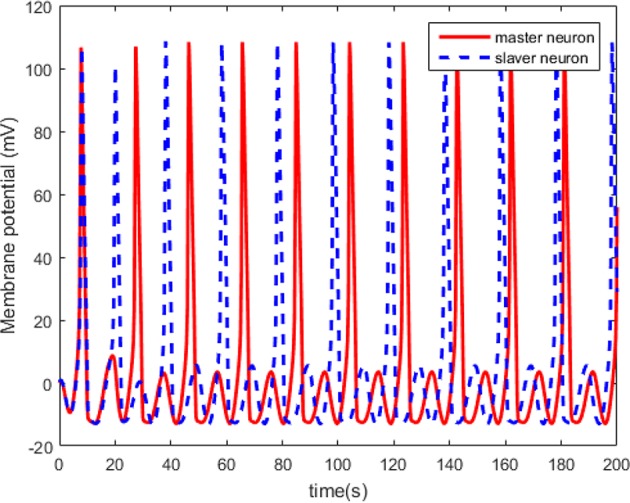
The original membrane potential curves of the master and the slaver HH neurons.

The simulations are performed on the master-slaver HH neuron system as Equation (3) and Equation (4), where $x_{M}={\left[x_{M,1},x_{M,2},x_{M,3},x_{M,4}\right]}^{T}$ is the system states of the master neuron with the initial conditions $x_{M}\left(0\right)={\left[0.1,0.2,0,0.2\right]}^{T}$, and $x_{S}={\left[x_{S,1},x_{S,2},x_{S,3},x_{S,4}\right]}^{T}$ is the system states of the master neuron with the initial conditions $x_{S}\left(0\right)={\left[0.3,0.1,0.2,0\right]}^{T}$. The specific parameters of the HH neuron models have been listed in Table 1. For the external stimulus current *I*_*ext*_*M*_ and *I*_*ext*_*S*_, the fixed parameters are listed in Table 2. The modulation frequencies of *I*_*ext*_*M*_ and *I*_*ext*_*S*_ are 100 and 104 Hz, respectively. Without external control, the original membrane potential curves of the master and the slaver HH neurons are shown in Figure 3. It is evident that the two HH neurons have different membrane potentials without external control.

The synchronization performance is predefined as follows. For the membrane potential synchronization error *e* of the two HH neurons, we expect that the minimum of convergence speed is greater than 0.6 s, the maximal steady-state error is <0.02, and the maximum of overshoot is <0.27%. Since the original condition *e*(0) = *x*_*S*,1_ − *x*_*M*,1_ = 0.2 > 0, the neural tracking error can be bounded by

(41) $$\begin{array}{l} -H\beta \left(t\right)<e<\beta \left(t\right) \end{array}$$

where *H* = 0.9, and

(42) $$\begin{array}{l} \beta \left(t\right)=\left(0.3-0.02\right)e^{-0.6t}+0.02 \end{array}$$

**Figure 4:**
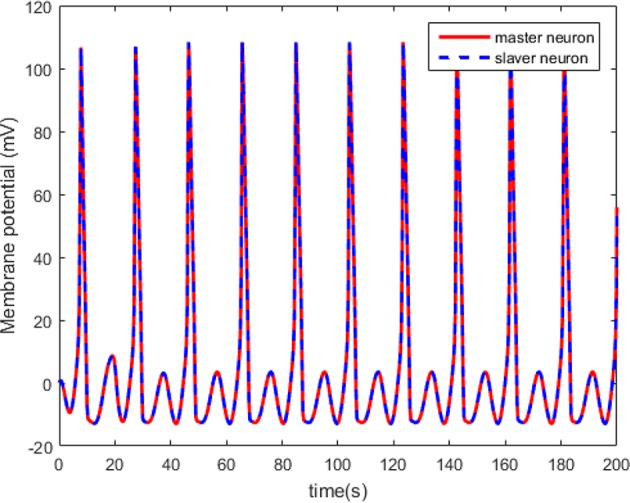
The synchronized membrane potential of the master and the slaver HH neurons.

**Figure 5:**
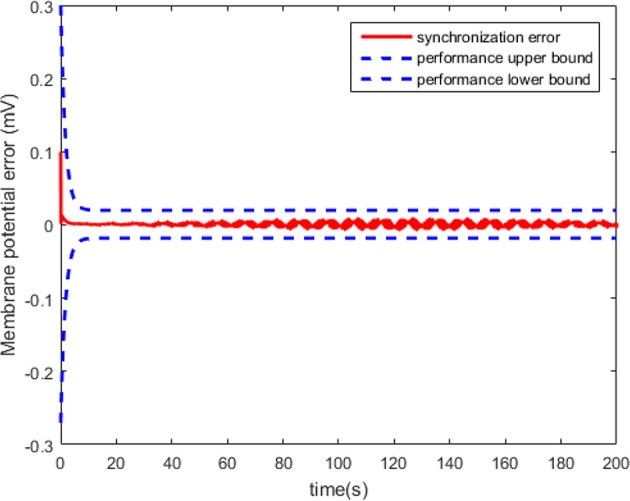
The synchronization error of the HH neuron system.

**Figure 6:**
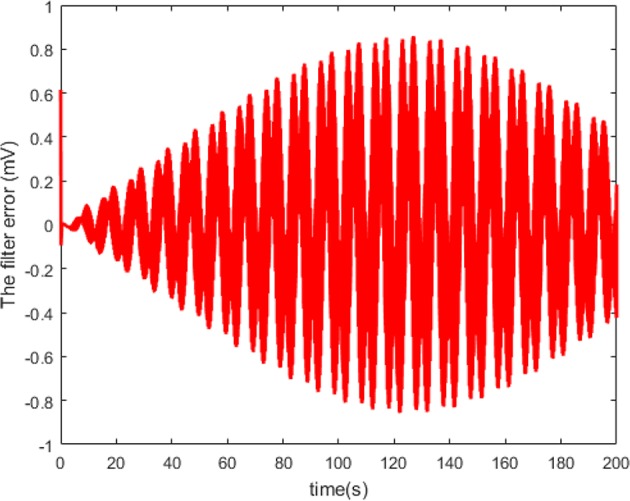
Curve of the filter error *ε*.

**Figure 7:**
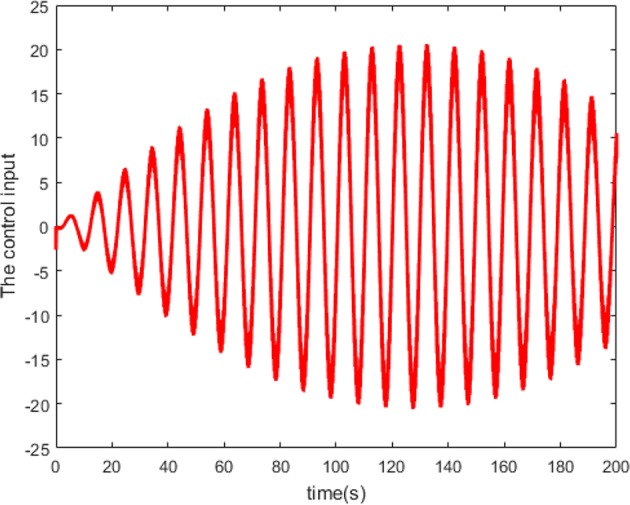
Control input with predefined performance.

According to Theorem 1, we design the synchronization controller and the adaptive tuning law of NN weight as (28), (29), and (30). Under the initial condition *e*(0) = 0.2 > 0, the filter tracking error ε and φ are defined in (22) and (25), respectively. In addition, we choice 441 nodes to structure the RBFNN Ŵ^*T*^*S*(*Z*), where the center μ is evenly distributed in [−2.5, 2.5], the width η = 0.25 and the initial weight Ŵ(0) = 0. The controller parameters are set as $k\left(t\right)=4-\dot{\varphi }/2\varphi^{2}$, *Q* = 1, and ξ = 0.001. The simulation results under the synchronization controller, which are satisfied the prescribed performance requirements, are shown in Figures 4–**7**.

The neuron state synchronization results are shown in Figures 4, 5. We can see that the slaver HH neuron quickly follows the master HH neuron, as shown in Figure 4. From Figure 5, it is obvious that the maximum overshoot of the synchronization error is <0.27% and the convergence rate is faster than the prescribed bound β(*t*) in (42) at the transient process. Furthermore, the results from Figure 5 also indicate that the synchronization error converges to zero exponentially, and the steady state error is <0.02. These results mean that the desired predefined performance synchronization is well accomplished. Figure 6 shows that the filter error ε is bounded. The control input *u* is presented in Figure 7.

Compared with the control laws in Octavio Cornejo-Pérez (2005) and Puebla et al. (2017), the superior synchronization performance of our adaptive neural controller is obviously revealed. For the sake of fairness, the same master-slaver HH neuron system with the same initial conditions is considered. Also, the same synchronization control performance is required. In Octavio Cornejo-Pérez (2005), an adaptive robust synchronization scheme to achieve robust synchronization is realized by introducing a feedback control law as follows:

(43) $$\begin{array}{l} u=\hat{\eta }+k{\hat{z}}_{1} \end{array}$$

and a high-gain observer is used to solve the problem of estimation (*z*_1_, η).

(44) $$\begin{array}{l} {\hat{z}}_{1}=\hat{\eta }-u+L_{0}k_{1}^{*}\left(z_{1}-{\hat{z}}_{1}\right) \end{array}$$

(45) $$\begin{array}{l} \hat{\eta }=L_{0}^{2}k_{2}^{*}\left(z_{1}-{\hat{z}}_{1}\right) \end{array}$$

The more parameters of (44) and (45) can be acquired in Octavio Cornejo-Pérez (2005).

**Figure 8:**
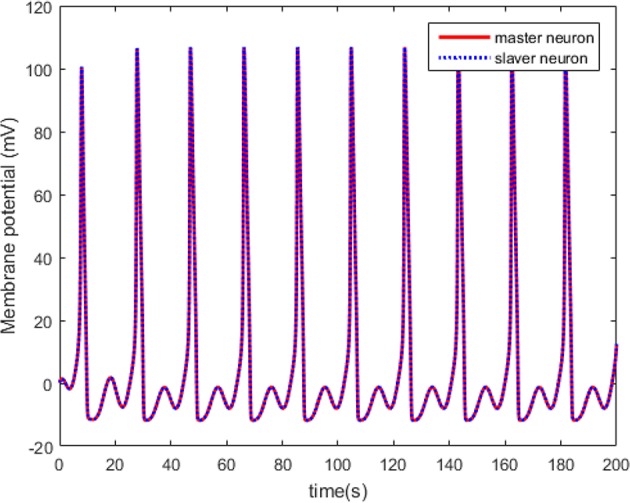
The synchronized membrane potential of the HH neurons with control law.

**Figure 9:**
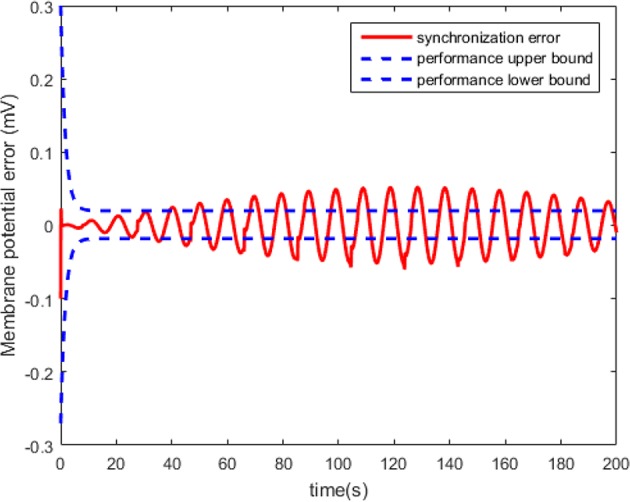
Synchronization error of the HH neuron system without predefined performance.

The results in Figures 8, 9 show that the adaptive scheme can make the HH neuron system reach robust synchronization of dynamical states. However, the synchronization error has higher amplitude than that under the proposed controller in this work (the dotted line represents the predefined bound of synchronization error).

In recent work (Puebla et al., 2017), a simple robust synchronization scheme for HH neural systems was represented by (46) and (47), based on a master-slaver configuration. The detailed parameters can be acquired in Puebla et al. (2017).

(46) $$\begin{array}{l} u\left(t\right)=-\tau_{c}^{-1}e\left(t\right)+\eta \left(t\right) \end{array}$$

(47) $$\begin{array}{l} \dot{\eta }\left(t\right)=-\tau_{e}^{-1}\left(\eta \left(t\right)-\bar{\eta }\left(t\right)\right) \end{array}$$

where τ_*c*_ and τ_*e*_ are the observer and synchronizer design parameters, respectively.

**Figure 10:**
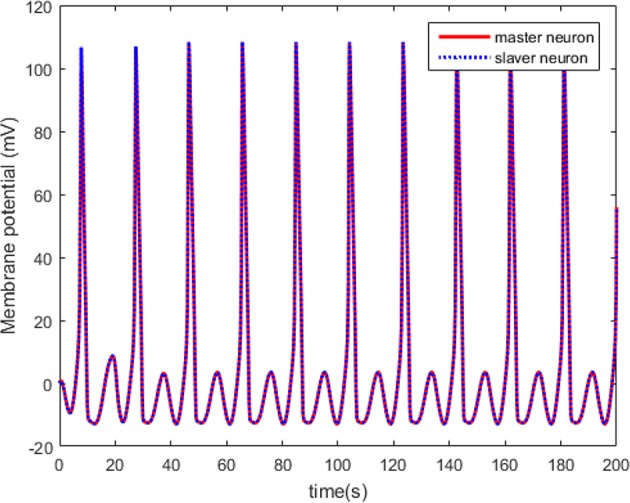
The synchronized membrane potential of the master and slaver HH neurons with control law.

**Figure 11:**
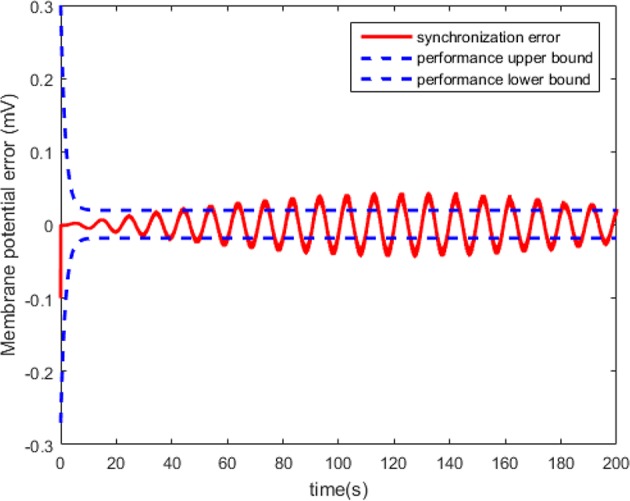
Synchronization error of the HH neuron system without predefined performance.

Figure 10 shows that the master and the slaver HH neurons arrive at synchronizing state quickly. However, the synchronization effect cannot be guaranteed as shown in Figure 11.

All of the above results demonstrate that the proposed adaptive neural controller can make the unidirectional coupled HH neurons under TMAS achieve state synchronization with superior synchronization effect, which satisfies the predefined performance.

## Discussion

In this paper, under TMAS, an adaptive neural controller is investigated for the prescribed performance synchronization of two dissimilar HH neurons connected through the medium of bidirectional coupling. A new transformation is introduced to make the equivalent unconstrained stabilization control problem instead of the constrained tracking problem. A stable synchronization controller is then designed by introducing a filter error into Lyapunov analysis. The proposed control laws overcome the uncertainties of the neuronal model and ensures the synchronization status of all the signals, as well as the prescribed synchronization performance in the closed-loop neuron system. Simulation results illustrate and verify the effectiveness of the proposed control mechanism.

In this work, we perform the simulation with single geometrical parameters of the cell. As we know, under the same stimulation conditions, there are different levels discharge of neurons with different cell geometrical parameters (Plant, 1976; Wang et al., 2003). Therefore, the discharge of neurons induced by TMAS may be changed when we alter the geometrical parameters of the cell.

TMAS has the advantages of noninvasive, high spatial resolution and high penetration depth. There are some limitations and disadvantages that need to be solved for TMAS in neuromodulation *in vivo*. (1) How the low-intensity ultrasound focus aims at the lesion location of the brain tissue. (2) How the static magnetic field distribution in the brain tissue needs to be further clarified. (3) Whether the current generated by TMAS in the brain tissue damages the neurons. Recently, Wang et al. used TMAS to modulate Parkinson's disease model mice (Wang et al., 2019). Their studies indicate that TMAS treatment improves the levels of brain-derived neurotrophic factor (BDNF), cAMP response element-binding protein (CREB), and protein kinase B (p-Akt) in the PD model mouse hippocampus. It demonstrates that TMAS can improve neuroplasticity in the hippocampus of Parkinson's disease model mice. Combined with the advantages and neuromodulation effects of TMAS, it has the potential to be used in the treatment and rehabilitation of neurological and psychiatric disorders.

Our study seeks to enhance the understanding of the processes that influence synchronization status of coupled neurons under TMAS in neuroses or psychoses. In biological neural network, thousands of neurons are interconnected under all kinds of intricate coupling phenomena. Thus, a large scale neural network has more complex dynamic characteristics and more difficult behavior to control than two connected neurons. The results in this study lay the basis for further synchronization research of Hodgkin-Huxley neuron network under TMAS. It can also provide theoretical guidance for practical applications of transcranial magneto-acoustical stimulation.

## Author Contributions

DL, XL, and YY designed and coordinated the study. DL, SZ, XL, and YY carried out data process and drafted the manuscript. All authors gave final approval for publication.

### Conflict of Interest

The authors declare that the research was conducted in the absence of any commercial or financial relationships that could be construed as a potential conflict of interest.
